# Sternal Puncture-Induced Acute Spinal Cord Syndrome in a Myeloma-Remissive Patient Revealing Pott Disease: A Case Report

**DOI:** 10.7759/cureus.58736

**Published:** 2024-04-22

**Authors:** Amina Er-rahmany, Klevor Raymond, Anselme Poda, Mohamed Chraa, Najib Kissani

**Affiliations:** 1 Neurology, Mohammed VI University Medical Center, Marrakesh, Marrakesh, MAR; 2 Nephrology, Nephrology and Dialysis Center of Marrakesh, Marrakesh, MAR

**Keywords:** hemodialysis, sternal puncture, magnetic resonance imaging, spinal cord, multiple myeloma, pott disease, tuberculosis

## Abstract

Cord compression is not a known complication of sternal puncture. We report the case of a patient with a history of multiple myeloma who presented acute onset paraplegia brought on during sternal puncture. Neuroimaging revealed focal spondylodiscitis and cord compression by an abscess. Neurosurgical decompression was not carried out on the patient because of her fragile general state of health and tardy consultation. Workup revealed the patient in remission from her multiple myeloma thus making decompressive radiotherapy unnecessary. The diagnosis of Pott disease was made by taking into account the clinical presentation, spine imaging and extra neurological imaging findings. Empiric anti-tuberculosis treatment was initiated which resulted in spectacular outcomes with a patient being able to walk with little aid by the end of her nine-month treatment course.

## Introduction

Acute spinal cord compression is a neurosurgical emergency [1]. It is a potential complication of Pott disease. Pott disease accounts for about 2% of all tuberculosis cases and 15% of extrapulmonary forms [2]. The frequency of cord compression due to spinal tuberculosis varies between 23 to 76% [3]. Tuberculosis is a global public health issue and remains endemic in many parts of the world. Patients with immunocompromised conditions due to cancers and immunosuppressant medication are particularly at risk of the infection which carries high morbidity and mortality [4].

We report the case of a patient with a history of multiple myeloma in remission who presented with subacute spontaneous back pain complicated with paraplegia during sternal puncture as the mode of revelation of Pott disease.

Sternal puncture is a procedure for sampling bone marrow for cytologic study. Though it is generally safe, rare complications may occur. The well-known complications involve injury to the pericardium, myocardium or mediastinal blood vessels which could be fatal [5]. However, to the best of our knowledge, no case has been reported yet of an acute spinal cord syndrome following this procedure.

## Case presentation

A 74-year-old patient with a history of multiple myeloma presented to the emergency department for acute onset paraplegia and urinary incontinence brought on by sternal puncture.

The diagnosis of multiple myeloma was made four years back and the patient had completed chemotherapy and was declared in remission. The patient was undergoing hemodialysis for chronic kidney disease and was receiving erythropoietin injections for anemia. She had a history of ischemic cardiomyopathy and was on anti-vitamin K therapy. Three months prior to presentation, the patient reported gradually worsening spontaneous back pain and constitutional symptoms of fatigue, night sweats, subjective weight loss and loss of appetite. Two months prior to presentation she underwent sternal puncture for follow-up of her hematologic state which revealed a patient in remission. However, the patient reported sudden worsening of back pain and paraplegia with urinary incontinence brought on by the sternal puncture.

On examination, the patient presented flaccid paraplegia, abolished deep tendon reflexes and Babinski sign bilaterally. Lasègue maneuver was unremarkable. Palpation of spinous processes of dorsal vertebrae worsened pain. Sensory function was preserved in the lower limbs. Examination of the upper limbs was unremarkable.

**Figure 1:**
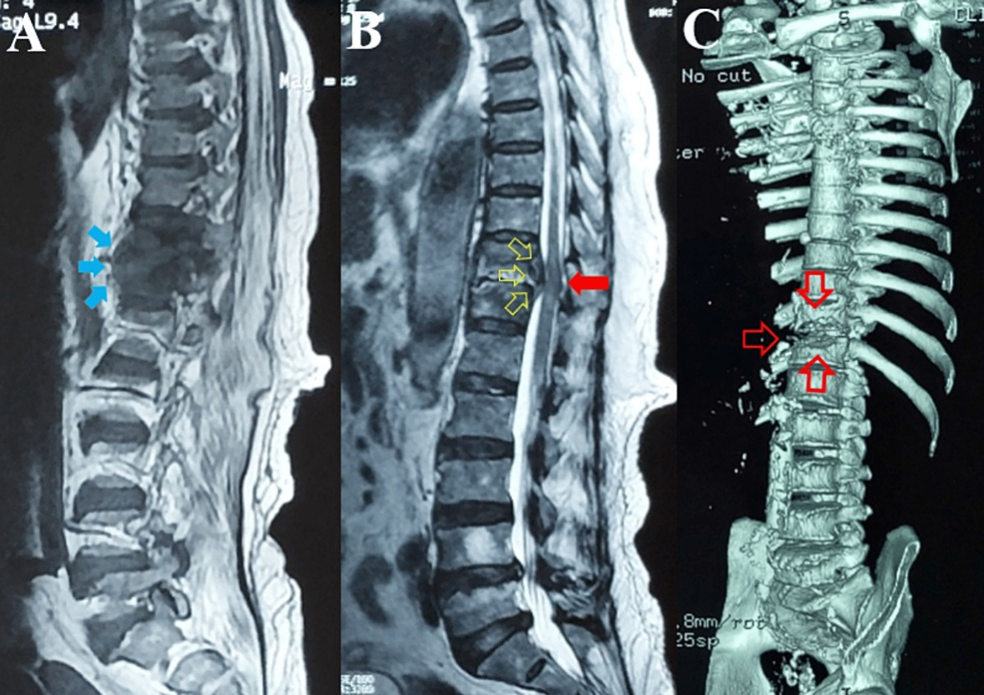
(A) T1 weighted Sagittal Spine MRI showing a pre and paravertebral abscess at the D11-D12 level (blue filled arrows). (B) T2 weighted Sagittal Spine MRI showing disk involvement at the D11-D12 level with a posterior wall abscess (hollow yellow arrows) and spinal cord compression (red filled arrow). (C) Computed tomography 3D reconstruction of the spine showing bone loss at the D11-D12 level (hollow red arrows).

On spine magnetic resonance imaging (MRI), findings were consistent with spondylodiscitis at the D11-12 level with paraspinal abscess resulting in focal cord compression (Figure 1).

Cerebrospinal fluid (CSF) analysis revealed isolated elevated protein of 0.76 g/l (normal range: 0.35-0.45 g/l) consistent with cord compression with normal cell count (<3/mm^3^) and CSF glucose level (0.51 g/l). Polymerase chain reaction (PCR) of the CSF was unremarkable. C-reactive protein was elevated (125 g/l) and QuantiFERON® was positive indicating probable *Mycobacterium tuberculosis* infection. Gene Xpert was negative. Complete blood count, serum and urinary proteins, and calcium assay were within norms. Blood cultures were negative. Bacteriology of sputum was also negative for *Mycobacterium tuberculosis*. Whole-body computed tomography (CT) scan revealed pulmonary nodules and micronodules, as well as bronchial dilation with calcified peritoneal nodules suggestive of multifocal tuberculosis.

Given the fragile physical state of the patient, surgical decompression was not performed. She was started on a nine-month regimen of anti-tuberculosis drugs: isoniazid, rifampicin, pyrazinamide, and ethambutol on days of dialysis (thrice weekly) and isoniazid and rifampicin on non-dialysis days for two months. This was followed by a seven-month course of daily isoniazid and rifampicin. After the first two months of medication and physical therapy, the patient showed spectacular recovery with a 3/5 force bilaterally in both lower limbs and a marked reduction of back pain. By the end of the nine-month course of treatment, the patient recovered a 5-/5 force in both lower limbs and was able to walk with minimal aid. She no longer presented back pain and had recovered a good general physical state. Follow-up was essentially clinical and imaging was not performed due to the financial obstacles of the patient.

## Discussion

Acute cord compression is a neurosurgical emergency [1,6]. Cord compression in our patient was likely due to fracture and posterior displacement of spondylitic bone or displacement of a cold abscess under the posterior longitudinal ligament with resulting conflict with the spinal cord [7]. Sternal puncture was the likely precipitating factor resulting in the loss of a fragile balance between the skeletal structures and the spinal canal. No mention exists of sternal puncture complicated by an acute spinal cord syndrome in the published literature, to the best of our knowledge. Known complications of the procedure include injury to the myocardium, pericardium, mediastinal blood vessels and pleura [5]. These complications result from direct injury of the penetrating needle to structures underlying the sternum.

Differential diagnoses for acute cord compression include trauma, disk herniation, malformations, neoplasms, and infections. In the context of multiple myeloma, cord compression could be due to infiltrative neoplasms, fracture of pathological bone, and abscess due to immune compromise. Patients with multiple myeloma have a higher risk of tuberculosis with greater morbidity [4]. Risk factors for Pott disease include malnutrition, alcoholism, diabetes mellitus, immunosuppressant medication use and human immunodeficiency virus (HIV) infection [8].

Pott disease is due to a hematogenous spread of *Mycobacterium tuberculosis* to vertebrae and disks from a primary site which could be genitourinary, abdominal, or pulmonary. The most frequent sites of spinal involvement are the thoracic and lumbar vertebrae which together constitute 80% to 90% of all spinal tuberculosis sites [9]. The clinical presentation includes back pain, spinal deformity, neurological impairment due to cord compression, and radiculopathy. Also, patients present with loss of weight, loss of appetite, fever, night sweats, and fatigue [2,3,7,9].

Management requires surgical decompression which should be carried out in patients with cord compression as this improves outcomes [6]. Ideally, decompression should be performed within 24 hours of symptom onset [1]. This was however not performed in our patient because of late presentation at the emergency and a fragile general state of health with serious health comorbidities. In case of compression by a neoplasm, radiotherapy is a viable option. However, surgery plus radiotherapy is superior to radiotherapy alone [10]. In the case of our patient, it was important to have an accurate diagnosis or at least exclude neoplasm in order to decide on the indication of radiotherapy since surgery was not an option for the patient. It helped that the results of the sternal puncture revealed the patient was in remission from multiple myeloma.

The diagnosis of tuberculosis is made based on proof of the existence of acid-fast bacteria from a biological sample of the patient. Samples include sputum, pus, CSF, and biopsied tissue. It is recommended to collect several samples sequentially in order to increase the sensitivity of tests [11]. Acid-fast bacilli (AFB) smear and mycobacterium culture allow for visualization of bacilli under light microscopy. Culture is superior to AFB smear but requires six to eight weeks for results. Gene Xpert however is a rapid method of diagnosing tuberculosis by amplification and detection of mycobacterium deoxyribonucleic acid (DNA) [7]. Given the severity of tuberculosis, the mortality of late initiation of treatment and its contagious nature, it is recommended to initiate treatment when there is a high clinical suspicion in high-risk patients without waiting for results of culture. This is especially useful in endemic areas like ours.

Given the context of immune compromise with multifocal lesions involving the spine, lungs, and peritoneum, the positive QuantiFERON and elevated C-reactive protein, with the absence of evidence of pyogenic bacteria in blood cultures, we had a high suspicion of tuberculosis and started our patient on empiric treatment with anti-tuberculosis drugs. A 4-drug regimen is recommended and the total duration of treatment is nine to 12 months [7]. The response to treatment in the case of our patient was another strong argument in favor of the diagnosis. The overall outcome was favorable for our patient.

## Conclusions

This case illustrates acute cord compression following fracture of a pathological bone during sternal puncture in a patient with a history of multiple myeloma as the mode of revelation of Pott disease. In endemic areas, it is important for the clinician to have a low threshold for diagnosis of tuberculosis especially in the context of immune compromise. Also, patients with immunocompromised conditions should be monitored for infectious complications. In case of a high index of suspicion, anti-tuberculosis therapy should be started on an empirical basis in order to reduce the morbidity and mortality associated with the infection.
